# Killing Bugs at the Bedside: A prospective hospital survey of how frequently personal digital assistants provide expert recommendations in the treatment of infectious diseases

**DOI:** 10.1186/1476-0711-3-22

**Published:** 2004-10-22

**Authors:** Steven D Burdette, Thomas E Herchline, W Scott Richardson

**Affiliations:** 1 Division of Infectious Disease, Department of Medicine, Wright State University School of Medicine, Dayton, Ohio 45409, USA; 2 Division of General Internal Medicine, Department of Medicine, Wright State University School of Medicine, Dayton, Ohio 45409, USA

## Abstract

**Background:**

Personal Digital Assistants (PDAS) are rapidly becoming popular tools in the assistance of managing hospitalized patients, but little is known about how often expert recommendations are available for the treatment of infectious diseases in hospitalized patients.

**Objective:**

To determine how often PDAs could provide expert recommendations for the management of infectious diseases in patients admitted to a general medicine teaching service.

**Design:**

Prospective observational cohort study

**Setting:**

Internal medicine resident teaching service at an urban hospital in Dayton, Ohio

**Patients:**

212 patients (out of 883 patients screened) were identified with possible infectious etiologies as the cause for admission to the hospital.

**Measurements:**

Patients were screened prospectively from July 2002 until October 2002 for infectious conditions as the cause of their admissions. 5 PDA programs were assessed in October 2002 to see if treatment recommendations were available for managing these patients. The programs were then reassessed in January 2004 to evaluate how the latest editions of the software would perform under the same context as the previous year.

**Results:**

PDAs provided treatment recommendations in at least one of the programs for 100% of the patients admitted over the 4 month period in the 2004 evaluation. Each of the programs reviewed improved from 2002 to 2004, with five of the six programs offering treatment recommendations for over 90% of patients in the study.

**Conclusion:**

Current PDA software provides expert recommendations for a great majority of general internal medicine patients presenting to the hospital with infectious conditions.

## 

PDAs (Personal Digital Assistants) are becoming widely used in medicine. A survey done by the America College of Physician predicted that 67% of physicians would be using PDAs by the end of 2002 [1]. These devices are used not only by the new generation of residents and physicians, but by all ages and all specialties [2,3]. PDA's are used not only as personal planners and contact lists but also for medical purposes such as patient billing and bedside medical references [4,5]. Physicians who have rapid and easy access to information are increasingly using that data when treating patients. This should ensure appropriate therapy and also reduce medical errors by ensuring proper dosing and treatment choices [6].

One medical specialty which has focused on PDA's application in medicine is infectious diseases [7]. There are numerous infectious disease programs which provide both expert recommendations regarding antibiotic choice as well as background information (epidemiology, diagnostic studies, source of pathogens, etc). We sought to determine the how often expert recommendations were available from the PDA infectious disease resources for the infectious conditions seen on our general internal medicine teaching service over a 4 month period of time. A secondary goal was to determine the change in the software over a 15 month period to provide expert recommendations.

## Methods

### Clinical Setting

Miami Valley Hospital is an 827 bed secondary and tertiary referral center, in Dayton, Ohio. It is an urban hospital that averages over three-thousand patient admissions per month. The internal medicine resident teaching service consists of two teams, each team with 2 senior residents and 2 interns who are supervised by an attending physician. There is a senior resident and intern on call in the hospital at all times. Patients were eligible to be admitted to resident's service if they were established within the Medical-Surgical Health Center of Miami Valley Hospital, if they were uninsured or if they had insurance but were without a local physician (a.k.a. private unattached patients). The general medicine teaching service averages approximately eight admissions per twenty-four hour period.

### Patients and Problems

The patients were screened prospectively over a four month period (July 2002 through October 2002). The admitting senior resident prospectively recorded the chief complaint and initial differential diagnosis for each patient. One of us (SDB), as the chief resident during these months, gathered the data during morning report. Patients were considered appropriate for the study if they had a leading diagnosis or active alternatives that suggested an infectious disease at the time of admission. Cases selected were then assigned categories based on the "major clinical syndromes" from Mandell, Douglass and Bennett's Principles and Practice of Infectious Disease (PPID) (8) (Table 1). Syndromes were categorized by organ system and were considered only if they would commonly require hospitalization (for example, sinusitis was not evaluated).

**Table 1:** Appropriate inpatient clinical syndromes according to PPID (8) and the distribution of patients admitted to the general medicine teaching service from July through October of 2002.

**Major Clinical Syndromes**	**Patients**
**Fever**	
Fever of Unknown Origin (Neutropenia)	6 (2)
**Upper Respiratory**	
Pharyngitis	2
Infections of head and neck	1
**Pleuropulmonary**	
Acute Bronchitis	16
Chronic Bronchitis	20
Acute Pneumonia	31
Empyema	2
Chronic Pneumonia	1
Cystic Fibrosis	1
**Urinary Tract**	22
**Sepsis Syndrome**	3
**Peritonitis and Other Intra-abdominal Infections**	3
**Cardiovascular**	
Endocarditis	2
**Central Nervous System**	
Acute Meningitis	6
Encephalitis	1
Brain Abscess	1
**Soft Tissue**	
Cellulitis and subcutaneous Tissue Infections	40
Lymphadenitis and Lymphangitis	2
**Gastrointestinal**	
Inflammatory Enteritides	20
Abdominal Symptoms and Fever	7
**Bone and Joint**	
Infectious Arthritis	1
Osteomyelitis	8
**STD**	
Prostatitis, Epididymitis and Orchitis	1
**Eye**	
Peri-ocular Infections	2
**HIV**	
Pulmonary Manifestations in HIV	5
CNS Manifestations in HIV	1

### Searching the PDA

A Sony Clie T615C (Palm OS) was the device used to access the software, but all programs evaluated were also available in the Pocket PC format (thus applicable to nearly 100% of PDAs in clinical use). Five PDA programs were initially searched to determine if expert recommendations were available. Software was chosen based on its availability to both the Palm OS and the Pocket PC and at the time of the initial software evaluation were the primary infectious disease programs available (since 2002 numerous other titles have been released to address infectious diseases). The PDA programs included: Sanford's Guide to Antimicrobial Therapy  (SG) [9], John's Hopkins Antibiotic Guide  (JHABx) [10], 5-Minute Infectious Diseases Consult ) (5MID) [11], 5-Minute Clinical Consult ) (5MCC) [12], and Pocket Medicine-Infectious Disease ) (PMID) [13]. The initial evaluation was performed with the most current software in October 2002 and reassessed in January 2004 with the latest versions of the available software. In addition, ePocrates ID ) (QID) [14] was also evaluated in January 2004 (but was not included in the October 2002 assessment as it was only available for the Palm OS at this junction).

A minimum of three attempts were used to locate conditions within each program. Searching was done using either the disease name or clinical problem that had led to the patient's admission. Synonyms were used when appropriate to increase the possibility of locating a condition within each program (for example: "urinary tract infection" was the first term searched and if no results available, then "pyelonephritis" was searched next and if still no results then "kidney infection" was entered into the database). Programs that were organized according to organ system (JHABx, QID only) were searched within the appropriate organ system, while programs that listed diagnoses alphabetically were searched accordingly. Expert recommendations were considered to be present and thus counted as a positive if the software had treatment recommendations present, whether related to antibiotic decision or "supportive care."

In order to determine the change in software capabilities over a 14 month period, the same patient data set was used to assess software programs in January of 2004. A comparison of the programs was then performed comparing the availability of expert recommendations.

### Statistical Analysis

The data was collected by a single physician (SDB) and then validated independently by a second physician (TEH). The data was analyzed using descriptive statistics with simple frequencies and the confidence intervals were calculated according to standard formulas.

## Results

**Figure 1:**
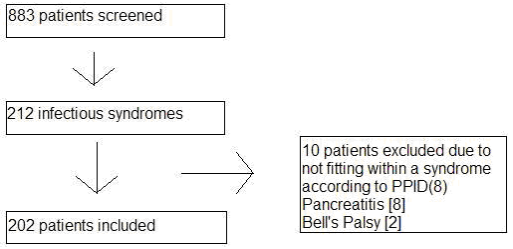
Results of patient screened for infectious etiologies over a 4 month period.

Over the four month period, from July through October of 2002, there were 883 patients admitted to the resident service, of whom 212 had syndromes that were suspected on admission to be infectious in etiology (202 of which could be assigned to the pre-defined categories) (See Figure 1 and Table 1).

As shown in Table 2, treatment recommendations were available in the PDA in at least one program for one-hundred percent of the patients admitted during the software evaluation in January 2004. Expert recommendations were available in all six of the programs for 52% of the patients admitted. The Sanford Guide and ePocrates ID each offered expert recommendations regarding treatment for 100% of the patients, while John's Hopkins Antibiotic Guide, 5 Minute Clinical Consultant and 5 Minute Infectious Diseases both offered expert recommendations in over 95% of the patients. Pocket Medicine-Infectious Disease offered expert recommendations for the fewest number of patients.

**Table 2:** Number and percent of patients with infection- related clinical syndromes covered by the studied PDA programs as of January 2004.

**Major Clinical Syndromes**	**Patients**	**SG**	**QID**	**JHABx**	**5MCC**	**5MID**	**PMID**	**Total % Rec**
**Fever**	6	6	6	6	4	4	4	100
**Upper Respiratory**	3	3	3	3	3	3	3	100
**Pleuropulmonary**	71	71	71	71	71	69	31	100
**Urinary Tract**	22	22	22	22	22	22	0	100
**Sepsis**	3	3	3	3	3	3	0	100
**Peritonitis**	3	3	3	3	3	3	2	100
**Cardiovascular**	2	2	2	2	2	2	2	100
**Central Nervous System**	8	8	8	8	8	8	7	100
**Soft Tissue**	42	42	42	42	42	42	40	100
**Gastrointestinal**	27	27	27	27	27	27	7	100
**Bone and Joint**	9	9	9	9	9	9	8	100
**STD**	1	1	1	0	0	1	0	100
**Eye**	2	2	2	2	2	0	2	100
**HIV**	6	6	6	6	0	0	0	100
**TOTALS**	202	202	202	201	195	192	106	202
**TOTAL %**		100	100	99	97	95	52	100
**95% Confidence Intervals**		100%	100%	97.5% to 100%	94.5% to 99.5%	92% to 98%	45% to 59%	

In regards to software changes between October 2002 and January 2004, each of the programs evaluated increased the number of patients for whom expert recommendations were available Table 3. The recommendations were not evaluated for changes in treatment over this time period, only the number of patients for whom recommendations were available was evaluated. The Sanford Guide had minimal improvements to make (initially offering expert recommendations for 96% of patients) while others improved significantly (JHABx improved from approximately 50% to over 96%). PMID, while offering treatment recommendations for the fewest number of patients, did show an improvement of 28% of the 14 month time period assessed. QID was assessed for the first time in 2004 when it was made available for both the Palm OS and Pocket PC and therefore no comparison data is available.

**Table 3:** Improvement in expert recommendations available in October 2002 as compared with January 2004.

**Programs**	**2002**	**2004**	**Difference**
**SG**	94%	100%	+6%
**QID**	NA	100%	NA
**JHABx**	61%	99%	+38%
**5MCC**	64%	97%	+33%
**5MID**	58%	95%	+37%
**PMID**	24%	52%	+28%

## Discussion

We were able to find expert recommendations regarding initial treatment of suspected infectious diseases on the PDA for 100% of patients admitted to the general medicine teaching service at Miami Valley Hospital over a 4 month period from July until October of 2002. As far as we know, this is the first ever prospective study of the breadth of coverage provided by PDA software for recommendations in infectious disease. Miller et al provide an excellent review and buyer's guide of many of the PDA software evaluated in this project and did sample the recommendations available for six selected infectious conditions [7].

This study should be interpreted in light of its potential limitations. First, we may have erred in our selection of which patients had infectious syndromes as the cause for admission. Patients' initial diagnoses might have been mistaken and the cause for admission was non-infectious. We have no information about how often such errors occurred. However, we attempted to assess the frequency of recommendations available for the initial diagnosis by the admitting physician rather than the discharge diagnosis.

A second potential limitation to our study is that searching errors occurred. Multiple steps were taken to limit the possibility that recommendations were credited that did not correctly match with the conditions sited or that recommendations were not found that did exist. Explicit criteria were used by an evaluator (SDB) familiar with both the PDA and the aforementioned software looking for management recommendations. This usually included antibiotic recommendations and occasionally recommendations regarding further evaluation. The possibility that we did not find recommendations which were indeed available seems unlikely because of the previously mentioned points as well as a systematic approach using multiple synonyms. Furthermore, the data was independently reviewed by a second physician and no inconsistencies were identified.

A third potential limitation is that of reproducibility. Since searching was done by an experienced user, searching errors would be more likely with less experienced user(s). Many of the programs require persistent use to learn the nuances and style of the programs and how to best access information. Therefore, in order for others to duplicate our results, users will need to develop some profiency with the software and be willing to perform searches using multiple synonyms.

Lastly, our study may have limits to its applicability. Our patients were admitted to an inpatient general internal medicine teaching service at an urban hospital in the United States. These data probably apply to similar inpatient services elsewhere within North America, but may not apply to either other specialty services (Pediatrics, Surgery, etc) or hospitals in other parts of the world.

While the limitations mentioned above apply to this study, they can also be expanded to include limitations in the software. No physician can access a software program for the first time and be expected to take full advantage of the recommendations available. It takes time and effort to maximize the clinical utility that is available on the PDA, which unfortunately, many clinicians do not accomplish. This paper was focused on treatment recommendations and did not evaluate the ability of the individual programs to assist with diagnosis. The diagnostic data and information other than treatment recommendations varies from program to program. This could be a topic for future research or may be available in other formats such as software reviews in PDA magazines.

This study demonstrates that these infectious disease programs have improved over time (table 3). The reasons for this are several. Many of the programs were newly released in 2002 and where advertised as a "works in progress." As time passed, they had time and resources to add more clinical data to their software (JHABx being the prime example) or they have released a more recent edition of the software (5MCC, 5MID, PMID). Secondly, the researcher (SB) had more time to become familiar with each program and therefore it is possible that part of the increased available recommendations is in part a "false positive" in that it was present in 2002 but not easily located (SG for example claims to have everything in the paperback version available in the PDA version, but accessing the data may be a challenge). This, as discussed previously, is an issue with software (often due to format or search engine) and increased experience with software definitely leads to increased clinical utility (both in regards to time and amount of information located). Table 3 also demonstrates the benefit of the "auto-update" feature that many of these programs possess. As new data is added to database, the device is able to access the central database and import these changes, thus making the software more dynamic rather than static. This allows the user the ability to use the latest recommendations, rather than material which may out of date. These features, allow with the compact size, mobility of the devices and the fact that multiple references may be available on a single device are just several examples of the benefit of using a PDA's in medicine.

Despite these potential limitations, our data suggests that PDAs provide expert recommendations for the majority of infectious clinical conditions encountered in practice by non-infectious disease general physicians. Since a majority of patients admitted to the hospital receive care from clinicians who are not specialists in infectious diseases, PDA's have the potential to have an impact on the quality of initial care by guiding not only the choice of antibiotics but also diagnostic testing and when to involve infectious disease specialists. Further research is needed to examine the efficiency of use; evidence supporting the recommendations, and the clinical impact of the use of these resources.
